# Expression of SARS-CoV-2-Related Surface Proteins in Non-Small-Cell Lung Cancer Patients and the Influence of Standard of Care Therapy

**DOI:** 10.3390/cancers14174074

**Published:** 2022-08-23

**Authors:** Christophe Deben, Maxim Le Compte, Vasiliki Siozopoulou, Hilde Lambrechts, Christophe Hermans, Ho Wa Lau, Manon Huizing, Kevin Lamote, Jeroen M. H. Hendriks, Peter Van Dam, Patrick Pauwels, Evelien L. J. Smits, Marc Peeters, Filip Lardon

**Affiliations:** 1 Center for Oncological Research (CORE), Integrated Personalized & Precision Oncology Network (IPPON), University of Antwerp, Universiteitsplein 1, B-2610 Wilrijk, Belgium; 2 Department of Pathology, Antwerp University Hospital, B-2650 Edegem, Belgium; 3 Multidisciplinary Oncologic Centre Antwerp (MOCA), Antwerp University Hospital, Wilrijkstraat 10, B-2650 Edegem, Belgium; 4 Biobank, Antwerp University Hospital, Wilrijkstraat 10, B-2650 Edegem, Belgium; 5 Laboratory of Experimental Medicine and Pediatrics, University of Antwerp, B-2610 Wilrijk, Belgium; 6 Internal Medicine and Pediatrics, Ghent University, B-9000 Ghent, Belgium; 7 Center for Cell Therapy and Regenerative Medicine, Antwerp University Hospital, B-2650 Edegem, Belgium; 8 Department of Oncology, Multidisciplinary Oncological Center Antwerp, Antwerp University Hospital, B-2650 Edegem, Belgium

**Keywords:** SARS-CoV-2, ACE2, lung cancer

## Abstract

**Simple Summary:**

SARS-CoV-2 is a respiratory virus that uses ACE2 for host cell entry and the spike protein is primed by, among others, TMPRSS2 and FURIN. The goal of this study was to determine in which non-small-cell lung cancer (NSCLC) patients these proteins are expressed on the membrane of the lung cancer cells and in which patients this increased ACE2 expression results in higher levels of soluble (s)ACE2 in their serum. In addition, we studied the influence of standard of care (SOC) therapies on sACE2 levels. Membranous (m)ACE2 was co-expressed with mFURIN and/or mTMPRSS2 in 16% of the NSCLC patients, and mACE2 and sACE2 were more frequently expressed in mutant EGFR patients but not mutant-KRAS patients. Importantly, systemic SOC therapies did not result in increased sACE2 levels. This indicates that cancer cells can be infected by SARS-CoV-2 in these patients, as well as that soluble ACE2 could impact the course of COVID-19.

**Abstract:**

In this study, we aimed to study the expression of SARS-CoV-2-related surface proteins in non-small-cell lung cancer (NSCLC) cells and identify clinicopathological characteristics that are related to increased membranous (m)ACE2 protein expression and soluble (s)ACE2 levels, with a particular focus on standard of care (SOC) therapies. ACE2 (*n* = 107), TMPRSS2, and FURIN (*n* = 38) protein expression was determined by immunohistochemical (IHC) analysis in NSCLC patients. sACE2 levels (*n* = 64) were determined in the serum of lung cancer patients collected before, during, or after treatment with SOC therapies. Finally, the TCGA lung adenocarcinoma (LUAD) database was consulted to study the expression of ACE2 in EGFR- and KRAS-mutant samples and ACE2 expression was correlated with EGFR/HER, RAS, BRAF, ROS1, ALK, and MET mRNA expression. Membranous (m)ACE2 was found to be co-expressed with mFURIN and/or mTMPRSS2 in 16% of the NSCLC samples and limited to the adenocarcinoma subtype. TMPRSS2 showed predominantly atypical cytoplasmic expression. mACE2 and sACE2 were more frequently expressed in mutant EGFR patients, but not mutant-KRAS patients. A significant difference was observed in sACE2 for patients treated with targeted therapies, but not for chemo- and immunotherapy. In the TCGA LUAD cohort, ACE2 expression was significantly higher in EGFR-mutant patients and significantly lower in KRAS-mutant patients. Finally, ACE2 expression was positively correlated with ERBB2-4 and ROS1 expression and inversely correlated with KRAS, NRAS, HRAS, and MET mRNA expression. We identified a role for EGFR pathway activation in the expression of mACE2 in NSCLC cells, associated with increased sACE2 levels in patients. Therefore, it is of great interest to study SARS-CoV-2-infected EGFR-mutated NSCLC patients in greater depth in order to obtain a better understanding of how mACE2, sACE2, and SOC TKIs can affect the course of COVID-19.

## 1. Introduction

Severe acute respiratory syndrome coronavirus 2 (SARS-CoV-2) is a respiratory virus for which the lungs are the primary site of infection. Consequently, the question of how this affects lung cancer patients who might be at risk of pulmonary complications from coronavirus disease arises. It has become clear that SARS-CoV-2 uses angiotensin I-converting enzyme 2 (ACE2) for host cell entry and that the spike protein of SARS-CoV-2 is primed by the cellular protease transmembrane serine protease 2 (TMPRSS2) and/or FURIN [1,2,3,4]. Consequently, cell populations with comparatively higher levels of ACE2 expression may be more vulnerable to SARS-CoV-2 infection [2]. Accordingly, Pinto et al. reported an increase in ACE2 mRNA expression in the lungs of patients with comorbidities associated with severe COVID-19 infections [5]. Importantly, they analyzed ACE2 expression from a lung cancer transcriptome dataset, showing that ACE2 was expressed in cancer samples at a higher level compared to adjacent normal lung tissue, which was later confirmed in other studies [5,6,7,8]. Consistent with the mRNA expression data, Feng et al. reported that among 64 samples of NSCLC tissues, 19 (29.6%) showed high ACE2 expression [9], and Yamaguchi et al. showed that tyrosine kinase inhibitor (TKI)-naïve EGFR-mutant lung adenocarcinomas mostly contain cancer cells expressing ACE2, to a greater extent than normal lung epithelia [10]. Importantly, Feng et al. reported that cisplatin and gemcitabine significantly upregulate ACE2 expression in A549 NSCLC cancer cells, indicating that standard of care (SOC) therapy can affect ACE2 expression levels in lung cancer cells in vitro [11]. For the latter, patient data are currently lacking, as well as data on the broad repertoire of SOC therapeutics for NSCLC. In addition, no reports on the co-expression of ACE2 and the TMPRSS2 and FURIN proteases required for effective SARS-CoV-2 infection were found. Therefore, we initiated this study to analyze the protein expression of ACE2, TMPRSS2, and FURIN on a panel of NSCLC patient tissue samples, taking into account neo-adjuvant therapies. In addition, we studied sACE2 levels in treatment-naïve and chemotherapy-, targeted-therapy-, or immunotherapy-treated NSCLC patients and malignant pleural mesothelioma patients to study if SOC therapies affect ACE2 levels in these patients. The exact role of sACE2 is still unclear, since it has both been explored as a potential therapy for COVID-19 [12] and been identified as a mediator of cell entry [13]. Finally, we studied the influence of SOC-targeted therapies on ACE2 protein expression levels in CALU-3 lung cancer spheroids.

## 2. Materials and Methods

### 2.1. FFPE Tissue Samples

Formalin-fixed paraffin-embedded (FFPE) tissue samples from 107 NSCLC patients and 1 colorectal adenocarcinoma metastasis to the lung were retrieved from the “Biobank Antwerp”, Antwerp, Belgium (ID: BE 71030031000) [14]. All the tissue samples were obtained through surgical resection as part of curative surgery, fixed in 4% formaldehyde for 6–18 h, and paraffin-embedded on a routine basis. This retrospective study was approved by the Ethics Committee of the Antwerp University Hospital/University of Antwerp (EC number 20/22/278). All samples were collected and archived before the COVID-19 pandemic. The patients’ clinicopathological characteristics are described in Table 1.

### 2.2. Serum Samples

A total of 64 serum samples from patients diagnosed with small-cell, non-small-cell lung cancer (MOCOR study), or malignant pleural mesothelioma (MesoBreath study) were obtained from the “Biobank Antwerp”, Antwerp, Belgium (ID: BE 71030031000) [14]. For a subset of patients, samples were collected during different phases of their disease and treatment schedule. In addition, 47 serum samples from healthy volunteers (non-cancer) were included in this study. These samples were collected in the oncology unit of the Antwerp University Hospital from the start of the pandemic as part of the MOCOR study described by Van Dam et al. [15]. The detection of SARS-CoV-2 in a subset of patients and healthy volunteers was performed using the Cobas SARS-CoV-2 test (Roche) on an automated CoBas 6800 system. Classically taken nose/throat samples were used as the gold standard for RT-PCR diagnosis. The use of these serum samples for this study was approved by the Ethics Committee of the Antwerp University Hospital/University of Antwerp (EC number 20/22/278). A detailed overview of the samples is provided in Supplementary Table S1 (showing diagnosis, sACE2 level, mutation status, treatment during sample collection, and SARS-CoV-2 status).

### 2.3. Immunohistochemical Staining

First, 5 µm-thick sections were prepared from FFPE specimens using a Microm HM 340E (Thermo Fisher Scientific, Waltham, MA, USA) microtome and the sections were placed on SuperFrost Plus (Thermo Fisher Scientific) slides. The slides were baked for 30 min at 60 °C to melt the paraffin. After that, they were hydrated through a series of xylene and graded isopropanol baths (100%, 95%, 70%, distilled water).

Sections were subjected to heat-induced antigen retrieval by incubation in a low-pH buffer (Envision Flex TRS low pH (DAKO)) for TMPRSS2 and in a high-pH buffer (Envision Flex TRS high pH (DAKO) for FURIN and ACE2 for 20 min at 97 °C (PT-Link, DAKO) in order to break the cross-links formed by formalin fixation. Slides were washed for 10 min in Envision Flex Wash buffer (DAKO) and an Immuno PEN was used to encircle the tissue. Staining was performed on a DAKO autostainer Link 48 using the Envision Flex detection kit. Endogenous peroxidase activity was quenched by incubation in peroxidase blocking buffer (DAKO) for 5 min. For ACE2, slides were first blocked with PBS + 2% Bovin Serum Albumin (Sigma) for 45 min to reduce background staining. The following primary antibodies were used: mouse anti-human ACE2 monoclonal antibody (R&D Systems, MAB933, 1:75 for 45 min), rabbit anti-human TMPRSS2 monoclonal antibody (Abcam, ab92323, 1:1000 for 30 min). and mouse anti-human FURIN (B-6) monoclonal antibody (Santa Cruz, sc-133142, 1:100 for 40 min). Primary antibody incubation was followed by a 30 min incubation with Envision FLEX/HRP (Ready to use, DAKO) secondary antibody and a 10 min incubation with the DAB substrate/chromogen detection system (DAKO). Between each incubation, the slides were washed twice in wash buffer (DAKO). The sections were counterstained for 2 min with hematoxylin (0.1%), dehydrated through a series of isopropanol baths (distilled water, 70%, 95%, 100%), cleared with xylene, and mounted with Quick-D Mounting Medium (KliniPath). Positive controls were included in each staining run and consisted of kidney tissue for ACE2 and TMPRSS2 and placenta tissue for FURIN (Figure S1). ACE2 and FURIN antibodies were validated for IHC by the Human Protein Atlas proteinatlas.org [16]; positive and negative controls are presented in Figure S1. An immunoreactivity scoring (IRS) system was applied. Staining intensity was designated as either no staining (0) or weak (+), moderate (++), or strong staining (+++), and the percentage of positive tumor cells was scored from 1 to 4 (<10%, 10–50%, 51–80%, and >80% of tumor cells stained). Afterwards, the overall IRS was calculated by the multiplication of these two parameters. Cases were grouped in four categories: no expression (0, IRS 0) and mildly (1, IRS 1–3), moderately (2, IRS 4–7), and strongly (3, IRS 8–12) positive.

### 2.4. ACE2 ELISA

SACE2 levels were determined using the RayBio Human ACE-2 ELISA kit. Serum samples were diluted 1/4.2 in the provided sample diluent and ELISA was performed according to the manufacturer’s instructions. Absorbance was measured at 450 nm on a Tecan Spark Cyto reader. Values (ng/mL) were interpolated from a standard curve using GraphPad Prism 9.

### 2.5. TCGA and Statistical Analysis

Gene mutation and mRNA expression (RSEM) data from The Cancer Genome Atlas (TCGA) Lung Adenocarcinoma (LUAD) PanCancer Atlas [17] cohort were extracted using cBioPortal [18,19]. An unpaired Student’s *t* test was used to study differences between two mutational groups. Co-expression Spearman’s correlation was determined using the co-expression module. The associations between clinicopathological parameters and protein expression levels were investigated by χ^2^ analysis for categorical variables using SPSS. The non-parametric Mann–Whitney U test was used to compare the sACE2 level means between the two groups using GraphPad Prism 9.

## 3. Results

### 3.1. ACE2, TMPRSS2 and FURIN Protein Expression on NSCLC Cells

**Figure 1:**
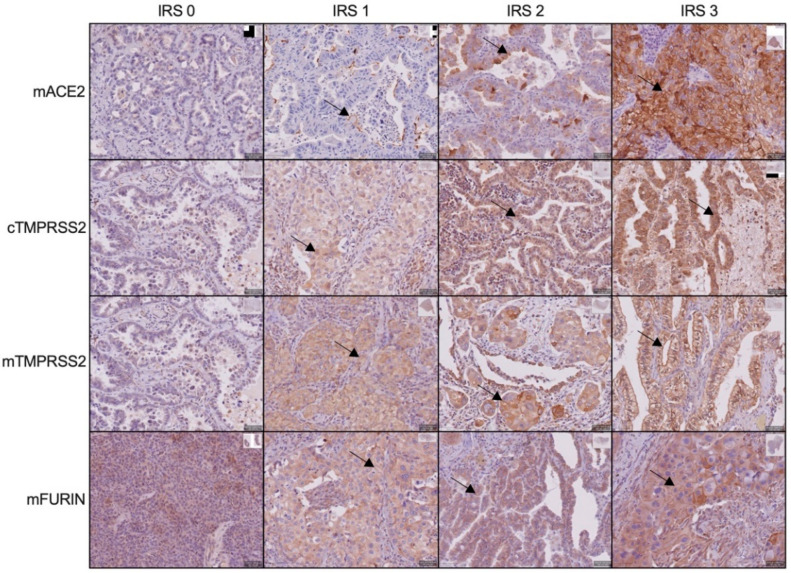
ACE2, TMPRSS2, and FURIN protein expression in NSCLC patients. Representative images of patient samples with an immune reactivity score (IRS) ranging from 0 to 3. m: membranous; c: cytoplasmic.

**Figure 2:**
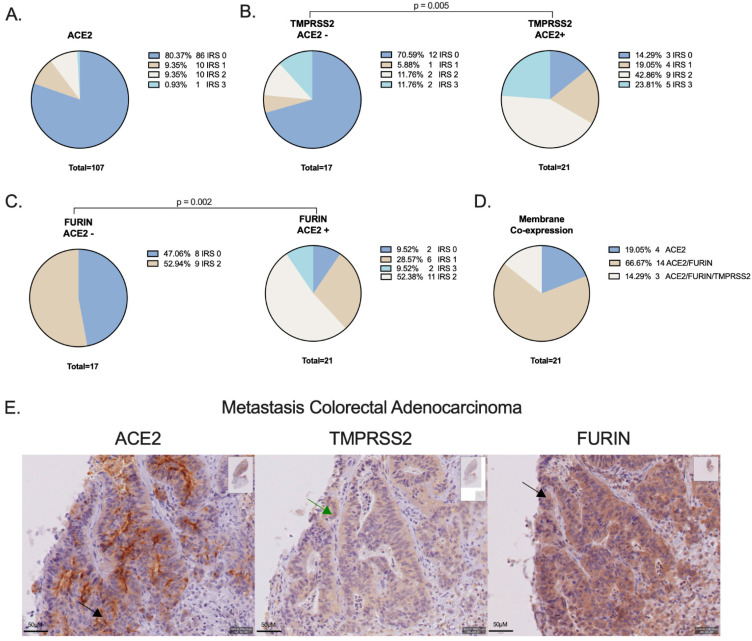
(**A**) Frequencies of ACE2 expression. (**B**) Frequencies of TMPRSS2 and (**C**) FURIN protein expression in ACE2− and ACE2+ patient samples. (**D**) Frequencies of the membranous co-expression of ACE2, TMPRSS2, and/or FURIN in the same tissue sample. (**E**) ACE2, TMPRSS2, and FURIN expression in a colorectal adenocarcinoma metastasis in the lung.

Figure 1 shows representative images for the different expression levels of membranous (m)ACE2, cytoplasmic (c)TMPRSS2, mTMPRSS2, and mFURIN. mACE2 expression was detected in 19.6% (21/107) of NSCLC patients, with 10% (11/107) showing a high expression level of IRS2 (10/107) or IRS3 (1/107)) (Figure 2A). Expression was not observed in the squamous cell carcinoma subtype (*n* = 7) and was limited to the adenocarcinoma subtype (*n* = 100) (Table 2). No association was observed between ACE2 expression (IRS0 vs. IRS1-3) and the patients’ clinicopathological data (gender, TNM classification, tumor stage, differentiation, smoker, PD-L1 expression, and KRAS or EGFR mutation) although a trend was observed for a higher frequency of ACE2 expression in EGFR-mutant patients (*p* = 0.063). Importantly, ACE2 expression was not increased in patients who received neo-adjuvant chemotherapy (cisplatin + pemetrexed/gemcitabine/etoposide/docetaxel/vinorelbine) (Table 2).

Since SARS-CoV-2 requires the co-expression of proteases TMPRSS2 and/or FURIN for infection, we determined their expression in a subset of patients (*n* = 38), including all ACE2+ patients (*n* = 21). TMPRSS2 was found to be significantly more highly expressed in ACE2+ versus ACE2− patient samples (86% vs. 29%, *p* = 0.005; Table 2; Figure 2B). Importantly, membranous TMPRSS2 expression was only observed in 22% (5/23) of the positive patients, showing that TMPRSS2 is atypically expressed in the cytoplasm (78%, 18/23). FURIN was significantly more highly expressed in ACE2+ versus ACE2− patient samples (95% vs. 53%, *p* = 0.002; Table 2; Figure 2C). Membranous expression was observed in 89% (25/28) of the FURIN-positive samples. ACE2 was co-expressed on the membrane with FURIN in 67% (14/21) of the samples and with FURIN/TMPRSS2 in 14% (3/21) of the samples. A total of 19% (4/21) of the ACE2+ samples showed no co-expression with the proteases (Figure 2D). Finally, ACE2 and FURIN expression was also observed in colorectal adenocarcinoma metastasis in the lung (Figure 2E).

### 3.2. sACE2 Levels

**Figure 3:**
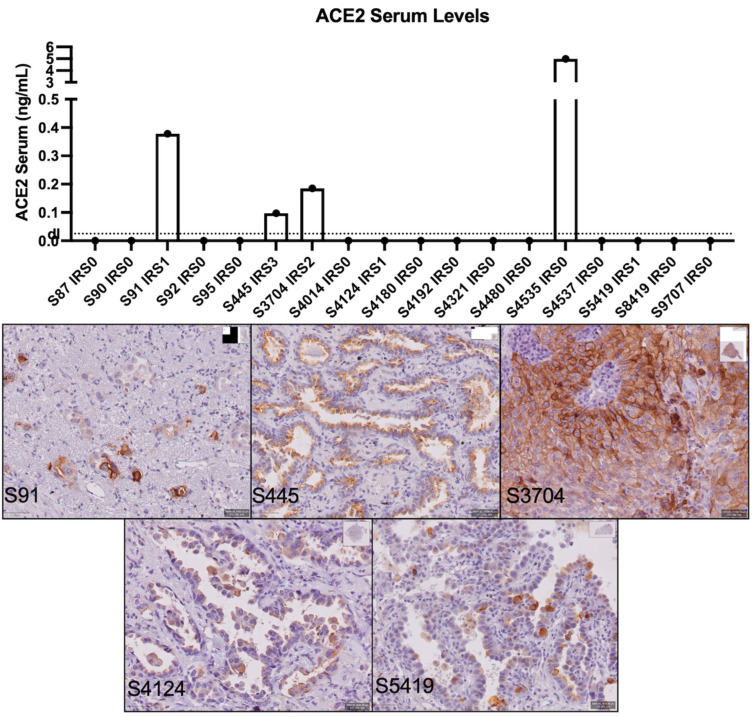
sACE2 levels and ACE2+ tumor samples.

sACE2 levels were quantified by ELISA in a subset of patients in which ACE2 protein expression was determined on the tumor (*n* = 18). Low levels of ACE2 could be detected in the blood of three patients with an IRS score ranging from 1 to 3 (Figure 3). ACE2 could not be detected in two patient samples with a low ACE2 expression on their tumor (IRS 1). All but one patient, who had a more than 10-fold higher concentration, with an IRS score of 0 showed no detectable levels of ACE2 in their blood. This indicates that quantifying ACE2 in the serum of NSCLC patients could be a valuable marker for identifying patients with high ACE2 expression on their tumor with a high specificity (91%) but limited sensitivity for low ACE2 expression (60%).

**Figure 4:**
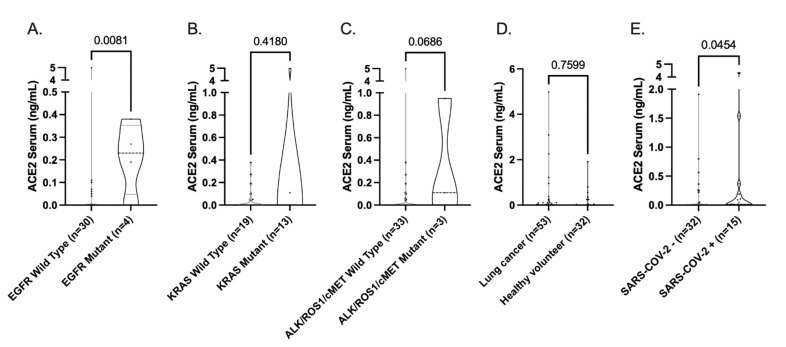
sACE2 levels. (**A**–**C**) sACE2 levels in NSCLC adenocarcinoma patients grouped by EGFR (*n* = 34), KRAS (*n* = 32), and ALK/ROS1/cMET mutation (*n* = 36). (**D**) Comparison of sACE2 levels between SARS-CoV-2 negative lung cancer patients and healthy volunteers. (**E**) Comparison of sACE2 levels between SARS-CoV-2-negative and -positive non-cancer volunteers. Differences in expression levels were determined by Mann–Whitney U statistical analysis, with *p* < 0.05 indicating significance.

Next, we expanded this NSCLC adenocarcinoma patient cohort with samples collected from the MOCOR study. Consistent with our IHC data, we observed a significantly higher concentration of ACE2 in the serum of EGFR-mutant versus wild-type patients (*p* = 0.0081), and a trend for ALK/ROS1/cMET mutant samples (*p* = 0.0680). No difference was observed for KRAS-mutant samples (*p* = 0.4180) (Figure 4A–C).

Lastly, we included a panel of healthy volunteers and RT-PCR-confirmed SARS-CoV-2-positive patients in our cohort. We observed no significant difference in sACE2 levels in our lung cancer populations (NSCLC, SCLC, MPM) compared to the healthy control population (Figure 4D). Interestingly, we observed significantly higher sACE2 levels in the SARS-CoV-2-positive subjects compared to the confirmed-SARS-CoV-2-negative subjects (Figure 4E).

### 3.3. Influence of Standard-of-Care Systemic Therapy on ACE2 Expression

**Figure 5:**
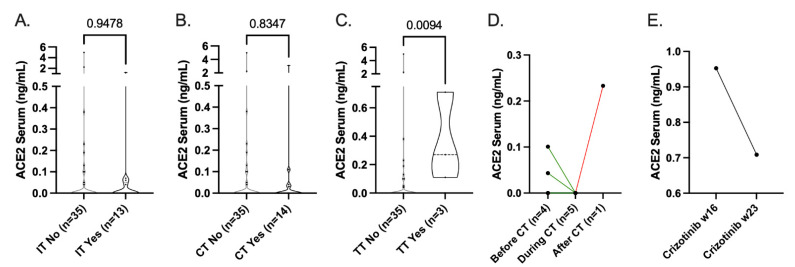
sACE2 levels related to systemic therapy. sACE2 levels in NSCLC adeno- and squamous-cell carcinoma, SCLC and malignant pleural mesothelioma lung cancer patients grouped by (**A**) immunotherapy (*n* = 13; Atezolizumab, Nivolumab, Pembroluzimab), (**B**) chemotherapy (*n* = 14; Carboplatin/Pemetrexed, Cisplatin/Pemetrexed, Carboplatin/Etoposide, Docetaxel), and (**C**) targeted therapy (*n* = 3; Osimertinib, Crizotinib). sACE2 levels of paired patient samples during different stages of (**D**) chemotherapy or (**E**) Crizotinib treatment. Differences in expression levels were determined by Mann–Whitney U statistical analysis, with *p* < 0.05 indicating significance. (IT: immunotherapy; CT: chemotherapy; TT: targeted therapy).

To study the potential influence of SOC systemic therapies on sACE2 levels, we further expanded our study cohort with other malignancies of the lung (small-cell lung cancer and malignant pleural mesothelioma (MPM)) collected for the MOCOR study. Neither immunotherapy (Atezolizumab, Nivolumab, Pembroluzimab; Figure 5A) nor chemotherapy (Carboplatin/Pemetrexed, Cisplatin/Pemetrexed, Carboplatin/Etoposide, Docetaxel; Figure 5B) affected the sACE2 levels in patients. When selecting samples of patients during different stages of their treatment, we observed a clear reduction in sACE2 levels during chemotherapy treatment (Carboplatin/Etoposide, Carboplatin/Pemetrexed, Cisplatin/Pemetrexed; Figure 5D). Patients treated with targeted therapy (Osimertinib and Crizotinib) had significantly higher levels of ACE2 in their serum (Figure 5C). One of these patients showed a decrease in sACE2 levels during the course of Crizotinib treatment (Figure 5E). Two out of three EGFR-mutant patients with detectable sACE2 levels did not receive treatment with TKIs prior to sample collection, suggesting that increased sACE2 levels are mutation-dependent rather than therapy-induced.

### 3.4. ACE2 Expression TCGA LUAD Cohort

**Figure 6:**
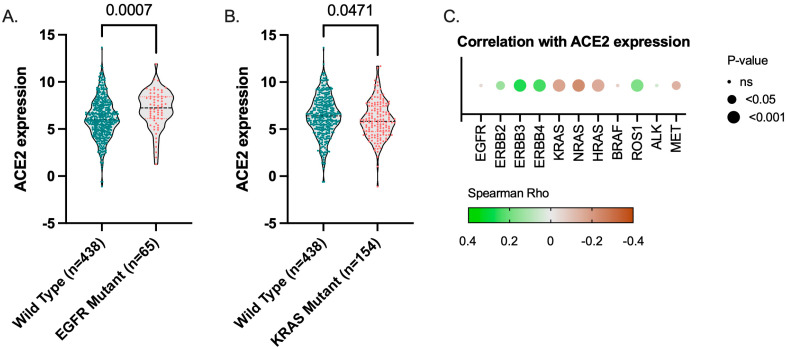
TCGA. ACE2 gene expression in (**A**) EGFR- and (**B**) KRAS-mutant TCGA LUAD tumors (RSEM, Log2). (**C**) Spearman’s correlation of ACE2 mRNA expression levels with EGFR/HER family members (EGFR, ERBB2-4), RAS (KRAS, NRAS, HRAS), BRAF, ROS1, ALK, and MET mRNA expression.

ACE2 mRNA expression is significantly increased in EGFR-mutant TCGA LUAD tumors (Figure 6A) and significantly decreased in KRAS-mutant tumors (Figure 6B). In addition, ACE2 expression is positively correlated with ERBB2, ERBB3, ERBB4, and ROS1 expression and negatively correlated with KRAS, NRAS, HRAS, and MET (Figure 6C). No correlation was found for EGFR, BRAF, and ALK expression levels.

## 4. Discussion

It has become clear that cancer patients are at higher risk of developing severe infection and mortality from COVID-19 or even of contracting an infection [20]. In a population-based nationwide Belgian cohort study, it was shown that hospitalized COVID-19-diagnosed cancer patients had a 34% higher chance of dying within 30 days of diagnosis compared with patients without cancer [21]. Multiple factors such as therapy-induced immunosuppression or co-existing medical conditions can attribute to a more severe course of COVID-19. Luo et al. have, for example, shown that patient-specific features such as smoking status and chronic obstructive pulmonary disease co-determined the severity of COVID-19 in lung cancer patients [22].

The goal of this study was to gain a better insight into the expression of the SARS-CoV-2-related proteins ACE2, TMPRSS2, and FURIN in lung cancer cells, making them susceptible to SARS-CoV-2 infection and potentially affecting the course of COVID-19. Furthermore, we studied sACE2 levels in different subsets of patients, particularly focusing on different treatment groups and driver mutations.

Consistent with the findings of Yamaguchi et al. [10], we found that ACE2 was frequently overexpressed in mutant EGFR lung adenocarcinomas, which was further supported by a significantly higher sACE2 concentration being found in these patients compared to wild-type EGFR samples. Interestingly, we observed no expression of ACE2 in mutant-KRAS patient samples, nor any difference in sACE2 levels. This suggests that the ACE2 expression in mutant EGFR patients is independent of MAPK pathway activation. Furthermore, Stewart et al. showed that higher ACE2 expression was associated with increased sensitivity to EGFR TKIs based on a drug constellation map from TCGA LUAD data, which further supports the relation between EGFR pathway activation and ACE2 expression [23]. Interestingly, Venkataraman et al. indicated that inhibiting EGFR signaling may also prevent excessive fibrotic response to SARS-CoV and other viral infections, since wound healing genes are regulated by EGFR signaling [24]. Therefore, SOC EGFR inhibitors could also alleviate COVID-19-related pulmonary fibrosis. Since we also observed higher sACE2 levels in patients with ROS1/cMET alterations, we hypothesize that ACE2 expression is dependent on PI3K pathway activation, which needs further validation. One patient receiving Crizotinib also showed reduced sACE2 levels during the course of his or her treatment. Importantly, a recent study identified mTOR/PI3K inhibitors, including Crizotinib, as ACE2-lowering compounds in cell lines, and patients who received these compounds had a statistically significantly lower rate of SARS-CoV-2 infection compared with patients given other antineoplastics [24]. Therefore, treatment with SOC TKIs in NSCLC could have a favorable outcome for these patients, as shown by case studies of patients receiving ALK/ROS1 inhibitors [25] or Osimertinib [26]. A limitation of this study is the low sample size of patients with ALK/ROS1/cMET alterations, preventing us from coming to a firm conclusion about ACE2 protein expression in these patients.

We found no indication that other classes of SOC therapies affect the expression of ACE2 in our patient cohort. This includes neoadjuvant chemotherapy for the FFPE samples and chemotherapy and immunotherapy for serum samples. Luo et al. report no impact of chemotherapy, PD-(L)1 blockade with or without chemotherapy, or TKIs on the severity of COVID-19 [22,27].

## 5. Conclusions

We show that ACE2 is co-expressed with membranous FURIN and/or TMPRSS2 in 16% of our NSCLC patient cohort, making these lung cancer cells susceptible to SARS-CoV-2 infection. Activating EGFR mutations were found to be associated with increased ACE2 expression, while no increased expression was observed in mutant-KRAS patients. SOC immunotherapy, chemotherapy, and targeted therapy did not increase sACE2 levels. Based on the literature, treatment with SOC TKIs could be beneficial for the course of COVID-19, which warrants further studies and follow-up evaluations of these subsets of SARS-CoV-2-infected lung cancer patients.

## Figures and Tables

**Table 1:** Clinicopathological characteristics.

Age (*n* = 107)	*n* (%)
≤65 years	55 (51.4)
>65 years	52 (48.6)
Gender (*n* = 107)	
Male	67 (62.6)
Female	40 (37.4)
T-classification (*n* = 106)	
T1	38 (35.8)
T2	37 (34.9)
T3	23 (21.7)
T4	8 (7.5)
N-classification (*n* = 107)	
N0	79 (73.8)
N1	17 (15.9)
N2	11 (10.3)
M-classification (*n* = 107)	
M0	100 (93.5)
M1	7 (6.5)
Tumor Stage (*n* = 106)	
I	49 (46.2)
II	29 (27.4)
III	21 (19.8)
IV	7 (6.6)
Subtype (*n* = 107)	
AC	100 (93.5)
SQ	7 (6.5)
Differentiation (*n* = 97)	
Poor	30 (30.9)
Moderate	37 (38.1)
Strong	30 (30.9)
Smoker (*n* = 89)	
No	8 (9)
Yes	81 (91.0)
Neoadjuvant Therapy (*n* = 107)	
No	85 (79.4)
Yes	22 (20.6)
EGFR Mutation (*n* = 56)	
No	47 (83.9)
Yes	9 (16.1)
KRAS Mutation (*n* = 22)	
No	13 (59.1)
Yes	9 (40.9)
PD-L1 (*n* = 23)	
≤1%	13 (56.5)
>1%	10 (43.5)
ACE2 (*n* = 107)	
IRS 0	86 (80.4)
IRS 1	10 (9.3)
IRS 2	10 (9.3)
IRS 3	1 (0.9)
TMPRSS2 (*n* = 38)	
IRS 0	15 (39.5)
IRS 1	5 (13.2)
IRS 2	11 (28.9)
IRS 3	7 (18.4)
FURIN (*n* = 38)	
IRS 0	10 (26.3)
IRS 1	6 (15.8)
IRS 2	20 (52.6)
IRS 3	2 (5.3)

**Table 2:** Patient clinicopathological data in relation to ACE2 protein expression.

	Membranous ACE2			Membranous TMPRSS2			Membranous Furin		
	IRS 0 (%)	IRS 1–3 (%)	*p*-Value	IRS 0 (%)	IRS 1–3 (%)	*p*-Value	IRS 0 (%)	IRS 1–3 (%)	*p*-Value
Age (*n* = 107, *n* = 38)									
≤65	45 (52.3)	10 (47.6)	0.443	17 (51.5)	2 (40)	0.631	7 (53.8)	12 (48.0)	0.732
>65	65 (47.7)	11 (52.4)		16 (48.5)	3 (60)		6 (46.2)	13 (52.0)	
Gender (*n* = 107, *n* = 38)									
Male	56 (65.1)	11 (52.4)	0.320	17 (51.5)	3 (60)	0.723	7 (53.8)	13 (52.0)	0.914
Female	30 (34.9)	10 (47.6)		16 (48.5)	2 (40)		6 (46.2)	12 (48.0)	
T-classification (*n* = 106, *n* = 37)									
T1	32 (37.6)	6 (28.6)	0.774	13 (40.6)	1 (20.0)	0.227	6 (50.0)	8 (32.0)	0.441
T2	30 (35.3)	7 (33.3)		8 (25.0)	1 (20.0)		1 (8.3)	8 (32.0)	
T3	17 (20)	6 (28.6)		6 (18.8)	3 (60.0)		3 (25.0)	6 (24.0)	
T4	6 (7.1)	2 (9.5)		5 (15.6)	0 (0.0)		2 (16.7)	3 (12.0)	
N-classification (*n* = 107, *n* = 38)									
N0	62 (72.1)	17 (81)	0.608	27 (81.8)	5 (100.0)	0.583	11 (84.6)	21 (84.0)	0.830
N1	14 (16.3)	3 (14.3)		4 (12.1)	0 (0.0)		1 (7.7)	3 (12.0)	
N2	10 (11.6)	1 (4.8)		2 (6.1)	0 (0.0)		1 (7.7)	1(4.0)	
M-classfication (*n* = 107, *n* = 38)									
M0	80 (93.0)	20 (95.2)	1.000	31 (93.9)	5 (100.0)	0.572	12 (92.3)	24 (96.0)	0.629
M1	6 (7.0)	1 (4.8)		2 (6.1)	0 (0.0)		1 (7.7)	1 (4.0)	
Tumor Stage (*n* = 106, *n* = 37))									
I	41 (48.2)	8 (38.1)	0.120	12 (37.5)	2 (40)	0.688	4 (33.3)	10 (40.0)	0.821
II	19 (22.4)	10 (47.6)		13 (40.6)	3 (60)		6 (50.0)	10 (40.0)	
III	19 (22.4)	2 (9.5)		5 (15.6)	0 (0.0)		1 (8.3)	4 (16.0)	
IV	6 (7.1)	1 (4.8)		2 (6.3)	0 (0.0		1 (8.3)	1 (4.0)	
Subtype (*n* = 107, *n* = 38))									
AC	79 (91.9)	21 (100)	0.341	30 (90.9)	3 (60.0)	0.057	9 (69.2)	24 (96.0)	**0.021**
SQ	7 (8.1)	0 (0.0)		3 (9.1)	2 (40.0)		4 (30.8)	1 (4.0)	
Differentiation (*n* = 97, *n* = 30))									
Poor	25 (31.6)	5 (27.8)	0.490	7 (28.0)	2 (40.0)	0.603	4 (36.4)	5 (26.3)	0.786
Moderate	28 (35.4)	9 (50.0)		14 (56.0)	3 (60.0)		6 (54.4)	11 (57.9)	
Strong	26 (32.9)	4 (22.2)		4 (16.0)	0 (0.0)		1 (9.1)	3 (15.8)	
Smoker (*n* = 89, *n* = 32)									
No	6 (8.7)	2 (10)	1.000	5 (17.2)	0 (0.0)	0.434	1 (10.0)	4 (18.2)	0.555
Yes	63 (91.3)	18 (90)		24 (82.8)	3 (100)		9 (90.0)	18 (81.8)	
Neoadjuvant Therapy (*n* = 107, *n* = 38)									
No	66 (76.7)	19 (90.5)	0.232	27 (81.8)	4 (80.0)	0.922	10 (76.9)	21 (84.0)	0.593
Yes	20 (23.3)	2 (9.5)		6 (18.2)	1 (20.0)		3 (23.1)	4 (16.0)	
EGFR Mutation (*n* = 56, *n* = 26)									
No	40 (88.9)	7 (63.6)	0.063	18 (75)	2 (100.0)	0.420	9 (81.8)	11 (73.3)	0.612
Yes	5 (11.1)	4 (36.4)		6 (25)	0 (0.0)		2 (18.2)	4 (26.7)	
KRAS Mutation (*n* = 22, n = 19))									
No	9 (50.0)	4 (100)	0.115	10 (58.8)	2 (100.0)	0.253	6 (66.7)	6 (60.0)	0.764
Yes	9 (50.0)	0 (0.0)		7 (41.2)	0 (0.0)		3 (33.3)	4 (40.0)	
PD-L1 (*n* = 23, *n* = 20)									
≤1%	10 (52.6)	3 (75.0)	0.604	11 (61.1)	1 (50.0)	0.761	6 (60.0)	6 (60.0	1.000
>1%	9 (47.4)	1 (25.0)		7 (38.9)	1 (50.0)		4 (40.0)	4 (60.0)	
TMPRSS2 (*n* = 38)									
IRS 0	12 (70.6)	3 (14.3)	0.005	N/A	N/A		6 (46.2)	9 (36.0)	0.618
IRS 1	1 (5.9)	4 (19.0)		N/A	N/A		2 (15.4)	3 (12.0)	
IRS 2	2 (11.8)	9 (42.9)		N/A	N/A		2 (15.4)	9 (36.0)	
IRS 3	2 (11.8)	5 (23.8)					3 (23.1)	4 (16.0)	
FURIN (*n* = 38)									
IRS 0	8 (47.1)	1 (4.8)	0.002	8 (24.2)	2 (40.0)	0.641	N/A	N/A	
IRS 1	0 (0.0)	7 (33.3)		6 (18.2)	0 (0.0)		N/A	N/A	
IRS 2	9 (52.9)	11 (52.4)		17 (51.5)	3 (60.0)		N/A	N/A	
IRS 3	0 (0.0)	2 (9.5)		2 (6.1)	0 (0.0)				

IRS: immunoreactivity scoring; N/A: not applicable.

## Data Availability

Data are available upon reasonable request.

## Supplementary Materials

The following are available online at https://www.mdpi.com/article/10.3390/cancers14174074/s1: Table S1: sACE2 concentrations and patient characteristics. Figure S1. IHC staining of control samples.
