# Supplementary material for: Expression of SARS-CoV-2-Related Surface Proteins in Non-Small-Cell Lung Cancer Patients and the Influence of Standard of Care Therapy
Source: Cancers (Basel). 2022 Aug 23;14(17):4074. doi: 10.3390/cancers14174074 (PMC9454734; doi:10.3390/cancers14174074)
Supplement: Supplementary file 1 [file cancers-14-04074-s001.zip › Table S1.pdf]

Table S1: sACE2 concentrations and patient characteristics

Subject-number	Diagnose	SARS-CoV-2	sACE2 ng/mL	ACE2 IHC	Treatment during sample collection	EGFR	KRAS	ALK/ROS/cMET	PIK3CA	PDL1	Cardio-vascular
S00000000087	LUAD	N/A	0.000	0	0	0	1	0	N/A	N/A	0
S00000000090	LUAD	N/A	0.000	0	0	0	1	0	N/A	N/A	0
S00000000091	LUAD	N/A	0.378	1	0	1	0	0	N/A	N/A	0
S00000000092	LUAD	N/A	0.000	0	0	0	1	0	N/A	N/A	0
S00000000095	LUAD	N/A	0.000	0	0	0	0	0	N/A	N/A	1
S00000000445	LUAD	N/A	0.097	3	0	0	0	0	N/A	N/A	1
S00000003704	LUAD	N/A	0.185	2	0	1	0	0	N/A	N/A	0
S00000004014	LUAD	N/A	0.000	0	0	0	1	0	N/A	N/A	0
S00000004124	LUAD	N/A	0.000	1	0	N/A	N/A	N/A	N/A	N/A	1
S00000004180	LUAD	N/A	0.000	0	0	0	0	0	N/A	N/A	1
S00000004192	LUAD	N/A	0.000	0	0	0	0	0	N/A	N/A	0
S00000004321	LUAD	N/A	0.000	0	0	0	0	0	N/A	N/A	0
S00000004480	LUAD	N/A	0.000	0	0	0	1	0	N/A	N/A	0
S00000004535	LUAD	N/A	4.989	0	0	0	1	0	N/A	N/A	0
S00000004537	LUAD	N/A	0.000	0	0	0	0	0	N/A	N/A	0
S00000005419	LUAD	N/A	0.000	1	0	0	0	0	N/A	N/A	0
S00000008419	LUAD	N/A	0.000	0	0	1	0	0	N/A	N/A	0
S00000009707	LUAD	N/A	0.000	0	0	0	0	1 (ALK)	N/A	N/A	0
S00000028540	LUAD	Negative	0.000	N/A	Pembroluzimab C26	0	1	0	N/A	90	0
S00000028540	LUAD	Negative	0.000	N/A	Pembroluzimab C28	0	1	0	N/A	90	0
S00000031111	MPM	N/A	0.000	N/A	0	N/A	N/A	N/A	N/A	N/A	0
S00000031111	MPM	N/A	0.000	N/A	Nivolumab C2D1	N/A	N/A	N/A	N/A	N/A	0
S00000031113	LUSC	Negative	0.000	N/A	Pembroluzimab C16	0	0	0	N/A	N/A	0

S00000031114	LUAD	Negative	0.000	N/A	0	0	1	0	N/A	N/A	1
S00000031114	LUAD	Negative	0.000	N/A	Carboplatin/Pemetrexed C2	0	1	0	N/A	N/A	1
S00000032988	MPM	Negative	0.000	N/A	Carboplatin/Pemetrexed D15	N/A	N/A	N/A	N/A	N/A	0
S00000032988	MPM	Negative	0.000	N/A	Nivolumab	N/A	N/A	N/A	N/A	N/A	0
S00000034883	LUAD	N/A	0.004	N/A	Cisplatin / Pemetrexed C2d1	N/A	N/A	0	0	N/A	1
S00000034969	MPM	N/A	0.000	N/A	0	N/A	N/A	N/A	N/A	N/A	1
S00000034969	MPM	N/A	0.000	N/A	Nivolumab C3	N/A	N/A	N/A	N/A	N/A	1
S00000035647	LUSC	Negative	0.709	N/A	Crizotinib	N/A	N/A	1 (cMET)	N/A	N/A	0
S00000035647	LUSC	Negative	0.953	N/A	Crizotinib	0	N/A	1 (cMET)	N/A	N/A	0
S00000037304	N/A	Negative	0.000	N/A	0	N/A	N/A	N/A	N/A	N/A	0
S00000038958	LUAD	Positive	0.000	N/A	0	0	1	0	N/A	90	1
S00000041919	LUAD	Negative	0.000	N/A	Pemetrexed	0	0	0	1	N/A	0
S00000058303	SCLC	Negative	0.000	N/A	Carboplatin/Etoposide/Atezolizumab	N/A	N/A	N/A	N/A	N/A	0
S00000058303	SCLC	Negative	0.043	N/A	0	N/A	N/A	N/A	N/A	N/A	0
S00000058413	LUAD	N/A	0.272	N/A	Osimertinib C2	1	0	0	N/A	N/A	0
S00000058628	MPM	Negative	0.000	N/A	Carboplatin/Pemetrexed C4D15	N/A	N/A	N/A	N/A	N/A	0
S00000058628	MPM	Negative	0.233	N/A	0 (before)	N/A	N/A	N/A	N/A	N/A	0
S00000058730	LUAD	Negative	0.000	N/A	Pembroluzimab C21	0	1	0	N/A	50	1
S00000059213	MPM	N/A	0.000	N/A	0	N/A	N/A	N/A	N/A	N/A	0
S00000061629	MPM	Negative	0.000	N/A	Cisplatin / Pemetrexed C2d1	N/A	N/A	N/A	N/A	N/A	0
S00000061629	MPM	Negative	0.101	N/A	0	N/A	N/A	N/A	N/A	N/A	0
S00000062513	MPM	Negative	3.091	N/A	Carboplatin/Pemetrexed	N/A	N/A	N/A	N/A	N/A	1
S00000064166	LUAD	Negative	0.000	N/A	Nivolumab	0	0	0	N/A	0	1
S00000064173	LUAD	Negative	0.000	N/A	Pemetrexed / Pembroluzimab C8	0	0	0	N/A	30	1
S00000064173	LUAD	Negative	0.000	N/A	Pemetrexed / Pembroluzimab C9	0	0	0	N/A	30	1
S00000068669	LUAD	Negative	0.000	N/A	Pembroluzimab C1D1	0	0	0	N/A	N/A	1

S00000075774	LUAD	Positive	0.112	N/A	Cisplatin/Pemetrexed	0	1	0	N/A	50	0
S00000075935	LUAD	Negative	0.029	N/A	Docetaxel	0	0	0	N/A	20	1
S00000075935	LUAD	Negative	0.036	N/A	Docetaxel	0	0	0	N/A	20	1
S00000075936	LUAD	Negative	0.072	N/A	Pembroluzimab C36	0	N/A	N/A	N/A	N/A	0
S00000075936	LUAD	Negative	0.042	N/A	Pembroluzimab C34	0	N/A	N/A	N/A	N/A	0
S00000078721	LUAD	Positive	0.045	N/A	0	0	0	0	0	90	0
S00000080975	LUAD	Negative	0.000	N/A	0	0	1	0	N/A	0	1
S00000084435	SCLC	Negative	1.218	N/A	Atezolizumab C17	N/A	N/A	N/A	N/A	N/A	0
S00000085306	LUAD	Negative	0.000	N/A	Pembroluzimab C20	0	1	0	N/A	50	1
S00000090185	MPM	Negative	0.000	N/A	0	N/A	N/A	N/A	N/A	N/A	0
S00000090185	MPM	Negative	0.000	N/A	Carboplatin/Pemetrexed C4D15	N/A	N/A	N/A	N/A	N/A	0
S00000091938	LUAD	Negative	0.062	N/A	Atezolizumab	0	N/A	0	N/A	N/A	1
S00000094876	LUAD	N/A	0.127	N/A	0	N/A	N/A	N/A	N/A	N/A	0
S00000096377	LUAD	N/A	0.107	N/A	Crizotinib	N/A	N/A	1 (ROS1)	N/A	N/A	0
S00000096787	N/A	N/A	2.253	N/A	0	N/A	N/A	N/A	N/A	N/A	1
S00000037600	HV	Negative	0.563	N/A	N/A	N/A	N/A	N/A	N/A	N/A	0
S00000037614	HV	Negative	0.000	N/A	N/A	N/A	N/A	N/A	N/A	N/A	0
S00000037616	HV	Negative	0.000	N/A	N/A	N/A	N/A	N/A	N/A	N/A	0
S00000037617	HV	Negative	0.004	N/A	N/A	N/A	N/A	N/A	N/A	N/A	N/A
S00000038065	HV	Negative	0.000	N/A	N/A	N/A	N/A	N/A	N/A	N/A	N/A
S00000038135	HV	Negative	0.004	N/A	N/A	N/A	N/A	N/A	N/A	N/A	N/A
S00000038250	HV	Negative	0.000	N/A	N/A	N/A	N/A	N/A	N/A	N/A	N/A
S00000038773	HV	Negative	0.000	N/A	N/A	N/A	N/A	N/A	N/A	N/A	N/A
S00000038777	HV	Negative	0.049	N/A	N/A	N/A	N/A	N/A	N/A	N/A	N/A
S00000038779	HV	Negative	0.000	N/A	N/A	N/A	N/A	N/A	N/A	N/A	N/A
S00000038780	HV	Negative	0.000	N/A	N/A	N/A	N/A	N/A	N/A	N/A	N/A

S00000038781	HV	Negative	0.258	N/A	N/A	N/A	N/A	N/A	N/A	N/A	N/A
S00000038782	HV	Negative	0.798	N/A	N/A	N/A	N/A	N/A	N/A	N/A	N/A
S00000038783	HV	Negative	0.000	N/A	N/A	N/A	N/A	N/A	N/A	N/A	N/A
S00000038787	HV	Negative	0.000	N/A	N/A	N/A	N/A	N/A	N/A	N/A	N/A
S00000038788	HV	Negative	0.000	N/A	N/A	N/A	N/A	N/A	N/A	N/A	N/A
S00000038792	HV	Negative	0.000	N/A	N/A	N/A	N/A	N/A	N/A	N/A	N/A
S00000038806	HV	Negative	0.000	N/A	N/A	N/A	N/A	N/A	N/A	N/A	N/A
S00000038815	HV	Negative	0.000	N/A	N/A	N/A	N/A	N/A	N/A	N/A	N/A
S00000038831	HV	Negative	0.361	N/A	N/A	N/A	N/A	N/A	N/A	N/A	N/A
S00000038835	HV	Negative	0.000	N/A	N/A	N/A	N/A	N/A	N/A	N/A	N/A
S00000038950	HV	Negative	1.911	N/A	N/A	N/A	N/A	N/A	N/A	N/A	N/A
S00000042572	HV	Negative	0.000	N/A	N/A	N/A	N/A	N/A	N/A	N/A	N/A
S00000052777	HV	Negative	0.248	N/A	N/A	N/A	N/A	N/A	N/A	N/A	N/A
S00000060884	HV	Negative	0.030	N/A	N/A	N/A	N/A	N/A	N/A	N/A	N/A
S00000064184	HV	Negative	0.000	N/A	N/A	N/A	N/A	N/A	N/A	N/A	N/A
S00000064215	HV	Negative	0.000	N/A	N/A	N/A	N/A	N/A	N/A	N/A	N/A
S00000066143	HV	Negative	0.000	N/A	N/A	N/A	N/A	N/A	N/A	N/A	N/A
S00000066348	HV	Negative	0.220	N/A	N/A	N/A	N/A	N/A	N/A	N/A	N/A
S00000066352	HV	Negative	0.000	N/A	N/A	N/A	N/A	N/A	N/A	N/A	N/A
S00000066353	HV	Negative	0.000	N/A	N/A	N/A	N/A	N/A	N/A	N/A	N/A
S00000094612	HV	Negative	0.000	N/A	N/A	N/A	N/A	N/A	N/A	N/A	N/A
S00000037617	HV	Positive	0.000	N/A	N/A	N/A	N/A	N/A	N/A	N/A	N/A
S00000038783	HV	Positive	0.012	N/A	N/A	N/A	N/A	N/A	N/A	N/A	N/A
S00000038784	HV	Positive	0.368	N/A	N/A	N/A	N/A	N/A	N/A	N/A	N/A
S00000038826	HV	Positive	0.046	N/A	N/A	N/A	N/A	N/A	N/A	N/A	N/A
S00000038830	HV	Positive	0.107	N/A	N/A	N/A	N/A	N/A	N/A	N/A	N/A

S00000038950	HV	Positive	4.328	N/A	N/A	N/A	N/A	N/A	N/A	N/A	N/A
S00000038957	HV	Positive	1.538	N/A	N/A	N/A	N/A	N/A	N/A	N/A	N/A
S00000039105	HV	Positive	0.015	N/A	N/A	N/A	N/A	N/A	N/A	N/A	N/A
S00000042572	HV	Positive	0.003	N/A	N/A	N/A	N/A	N/A	N/A	N/A	N/A
S00000064215	HV	Positive	0.002	N/A	N/A	N/A	N/A	N/A	N/A	N/A	N/A
S00000066149	HV	Positive	0.000	N/A	N/A	N/A	N/A	N/A	N/A	N/A	N/A
S00000066348	HV	Positive	0.193	N/A	N/A	N/A	N/A	N/A	N/A	N/A	N/A
S00000084442	HV	Positive	0.000	N/A	N/A	N/A	N/A	N/A	N/A	N/A	N/A
S00000085016	HV	Positive	0.090	N/A	N/A	N/A	N/A	N/A	N/A	N/A	N/A
S00000085086	HV	Positive	0.000	N/A	N/A	N/A	N/A	N/A	N/A	N/A	N/A

LUAD: Lung Adenocarcinoma; LUSC: Lung Squamous Cell Carcinoma; MPM: Malignant Pleural Mesothelioma; HV: Healthy Volunteer; N/A: Not Applicable; C: Cycle; D; Day. Treatment during sample collection. SARS-CoV-2 status during sample collection
