# Supplementary figures and images for: Expression of SARS-CoV-2-Related Surface Proteins in Non-Small-Cell Lung Cancer Patients and the Influence of Standard of Care Therapy
Source: Cancers (Basel). 2022 Aug 23;14(17):4074. doi: 10.3390/cancers14174074 (PMC9454734; doi:10.3390/cancers14174074)

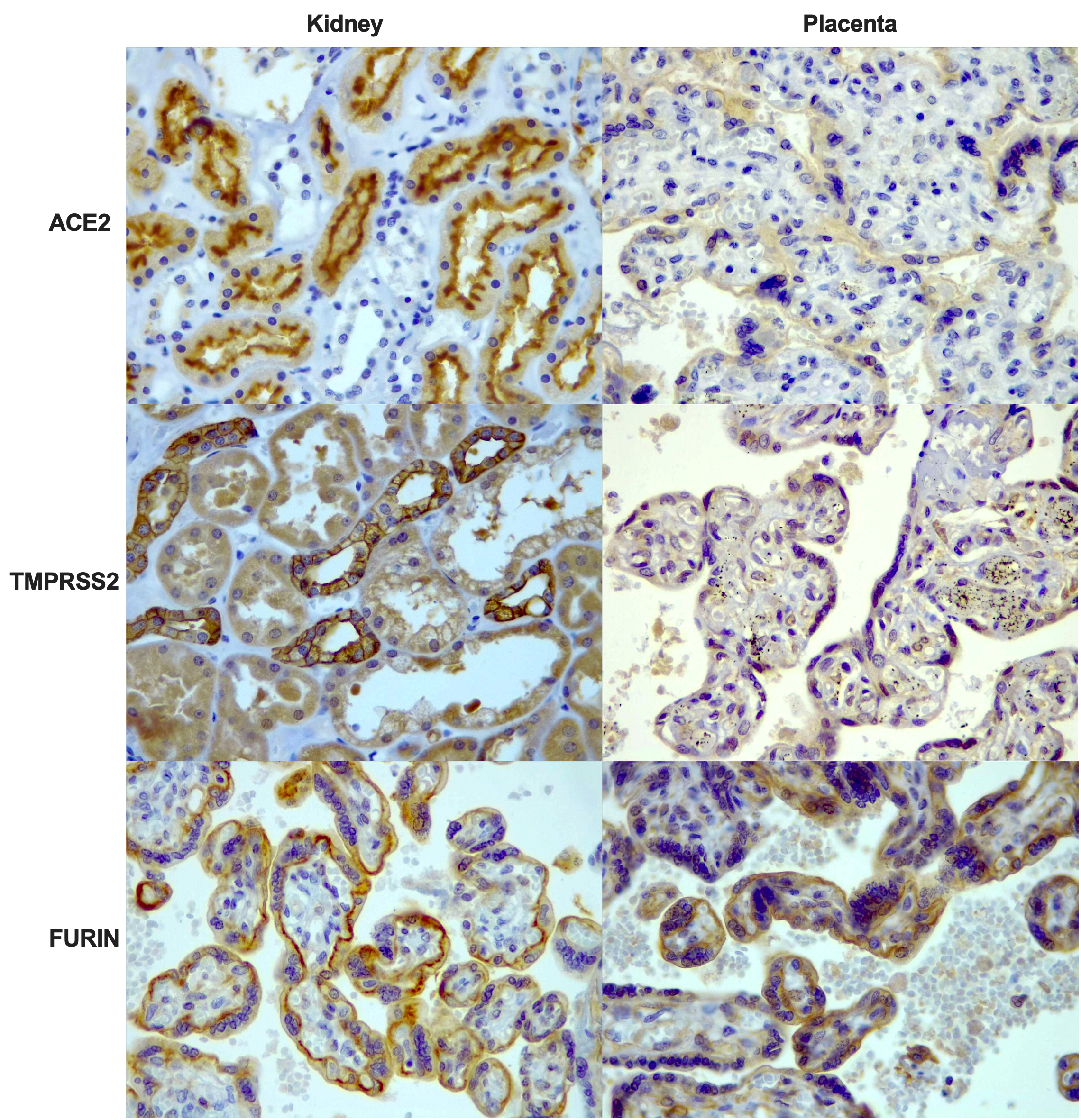

Supplement: Supplementary file 1 [file cancers-14-04074-s001.zip › FigureS1.png]
